# In vitro surgical and non-surgical air-polishing efficacy for implant surface decontamination in three different defect configurations

**DOI:** 10.1007/s00784-020-03476-1

**Published:** 2020-08-19

**Authors:** Vivian Tuchscheerer, Peter Eickholz, Bettina Dannewitz, Christoph Ratka, Otto Zuhr, Hari Petsos

**Affiliations:** 1 Salzhausen, Germany; 2 Department of Periodontology, Center for Dentistry and Oral Medicine (Carolinum), Johann Wolfgang Goethe-University Frankfurt/Main, Theodor-Stern-Kai 7, 60596 Frankfurt/Main, Germany; 3 Weilburg, Germany; 4 Department of Prosthodontics, Center for Dentistry and Oral Medicine (Carolinum), Johann Wolfgang Goethe-University Frankfurt/Main, Theodor-Stern-Kai 7, 60596 Frankfurt/Main, Germany; 5 Munich, Germany; 6 Soest, Germany

**Keywords:** Dental implants, Decontamination, Abrasion, Dental air, Peri-implantitis

## Abstract

**Objectives:**

Evaluation of surgical and non-surgical air-polishing in vitro efficacy for implant surface decontamination.

**Material and methods:**

One hundred eighty implants were distributed to three differently angulated bone defect models (30°, 60°, 90°). Biofilm was imitated using indelible red color. Sixty implants were used for each defect, 20 of which were air-polished with three different types of glycine air powder abrasion (GAPA1–3) combinations. Within 20 equally air-polished implants, a surgical and non-surgical (with/without mucosa mask) procedure were simulated. All implants were photographed to determine the uncleaned surface. Changes in surface morphology were assessed using scanning electron micrographs (SEM).

**Results:**

Cleaning efficacy did not show any significant differences between GAPA1–3 for surgical and non-surgical application. Within a cleaning method significant (*p* < 0.001) differences for GAPA2 between 30° (11.77 ± 2.73%) and 90° (7.25 ± 1.42%) in the non-surgical and 30° (8.26 ± 1.02%) and 60° (5.02 ± 0.84%) in the surgical simulation occurred. The surgical use of air-polishing (6.68 ± 1.66%) was significantly superior (*p* < 0.001) to the non-surgical (10.13 ± 2.75%). SEM micrographs showed no surface damages after use of GAPA.

**Conclusions:**

Air-polishing is an efficient, surface protective method for surgical and non-surgical implant surface decontamination in this in vitro model. No method resulted in a complete cleaning of the implant surface.

**Clinical relevance:**

Air-polishing appears to be promising for implant surface decontamination regardless of the device.

## Introduction

There is clear evidence that plaque is the primary etiological factor for the development of peri-implant mucositis or peri-implantitis [1] as it was compiled at the 2017 classification workshop for periodontal and peri-implant diseases [2]. Thus, prevention and therapy of these diseases aims to disrupt the biofilm on the implant surface as a cause. In addition to an ineffective plaque control [3], there are other risk factors such as smoking [4], history of periodontitis [5, 6], and irregular maintenance [7, 8]. Non-surgical treatment of peri-implant mucositis has turned out to be the method of choice [9–11], while in peri-implantitis non-surgical treatment alone is largely not effective [1, 9, 12] due to the re-maturing of plaque. In contrast, the surgical intervention with direct insight to the defect, better accessibility, and the possibility to avoid recolonization due to resective or regenerative procedures has proven to be superior [13, 14]. Nevertheless, the implant geometry with its micro- and/or macro-threads and the rough surface morphology with an individual surface roughness remain difficult to clean in daily routine even with good insight and improved accessibility [15, 16]. Rigid instruments such as curettes and (ultra)sonic scalers with steel tips have recently failed to convince in both non-surgical and surgical simulated in vitro experiments [17–19]. Although these in vitro results seem clear, current clinical evidence does not show clear data for or against the use of these instruments [20]. Alternatives such as lasers and air-polishing systems partly offer beneficial results when used on complex implant surfaces [21, 22]. However, this benefit seems to be limited depending on defect morphology and extension [9, 20, 23]. Using the laser, this limitation additionally seems to be due to its rigid tip, which impairs the application of the laser beam, and the consequently poorer accessibility [24]. When using air-polishing, the powder particles have the advantage of being able to be reflected in the typically shaped peri-implant bone defects, which probably leads to a cleaning effect even in areas that are difficult to access [17, 25]. The use of glycine-based powder, which is a human protein component, has the advantage of being absorbable and not remaining in a wound as a foreign body. On the other hand, scanning electron microscope (SEM) images have recently shown that glycine-based powder has a less abrasive effect on implant surfaces compared to sodium bicarbonate powder [26–28].

The use of air-polishing with glycine powder has proven to be superior in both the surgical [17, 19] and the non-surgical [18] in vitro approach compared to steel curettes and (ultra)sonic scaler with steel tips. Consequently, this study aims to compare different air-polishing methods in both approaches for three defect configurations [30°, 60° (intraosseous defects), 90° (supraosseous defect)] in order (i) to expand the available data on efficacy of surgical and non-surgical air-polishing application and (ii) to compare the efficacy of different air-polishing devices in both, a surgical and non-surgical in vitro simulation. The null hypothesis was that all air-polishing device combinations showed statistically different outcomes in terms of percentage of color remnants on the implant surface according to the type of approach and defect morphology and that different surface alterations resulted after cleaning. To the best of the authors’ knowledge, no further in vitro investigation is known that compares the efficacy of different glycine-based air powder abrasion devices for implant surface decontamination.

## Materials and methods

### Implant preparation and model

The study was largely based on the set-up by Ronay et al. [18] and also follows approaches from Sahrmann et al. [19] and Keim et al. [17].

One hundred and eighty tissue-level implants with a subcrestal length of 12 mm and a diameter of 4.1 mm (OKTAGON^®^ Dental Implant System—made by Meisinger, DENTAL RATIO^®^, Langenfeld, Germany) were dipped for 5 s into red color (Staedler permanent lumocolor, Nuremberg, Germany) and air-dried for 24 h to imitate a plaque-covered surface. All parts of the surface were completely and homogeneously covered. The implant consisted of a 2.3-mm machined surface in the supracrestal part and a rough sand-blasted and etched surface with a mean surface roughness of 1.69 μm in the subcrestal part. The macrostructure of the rough implant surface was made of 0.35mm high threads arranged at a distance of 1.25 mm from each other.

**Fig. 1:**
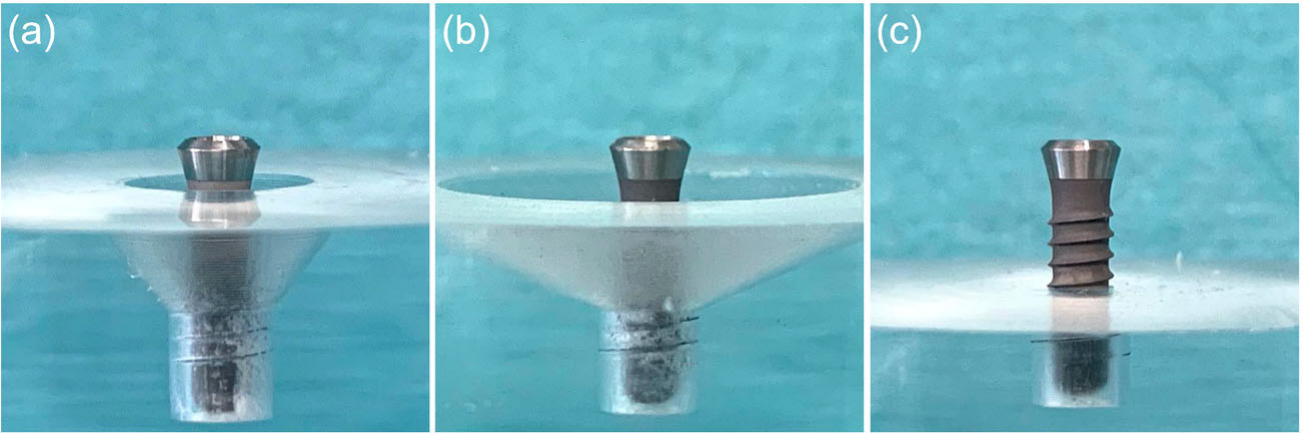
Lateral view of the three different defect angulations **a** 30°, **b** 60°, and **c** 90° without mucosal mask

**Fig. 2:**
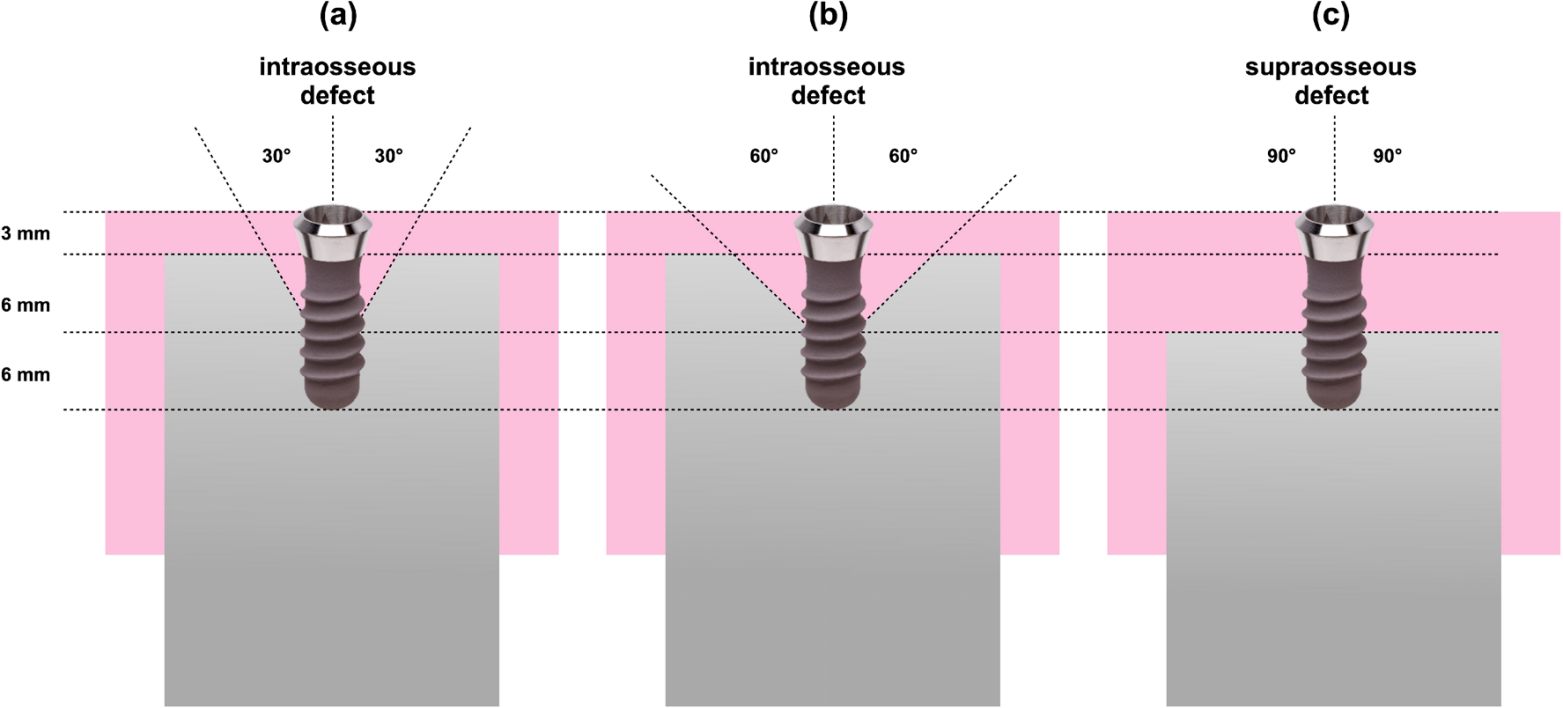
Drawings of the three different defect models **a** 30°, **b** 60°, and **c** 90° with mucosal mask each with an exemplary inserted schematic draft of an untreated OKTAGON 4.1 × 12 mm (OKTAGON^®^ Dental Implant System—made by Meisinger, DENTAL RATIO^®^, Langenfeld, Germany) implant with 0.35 mm thread depth and 1.25 mm thread distance (macro-thread). A gingival thickness of 3 mm was simulated in the non-surgical approach

**Fig. 3:**
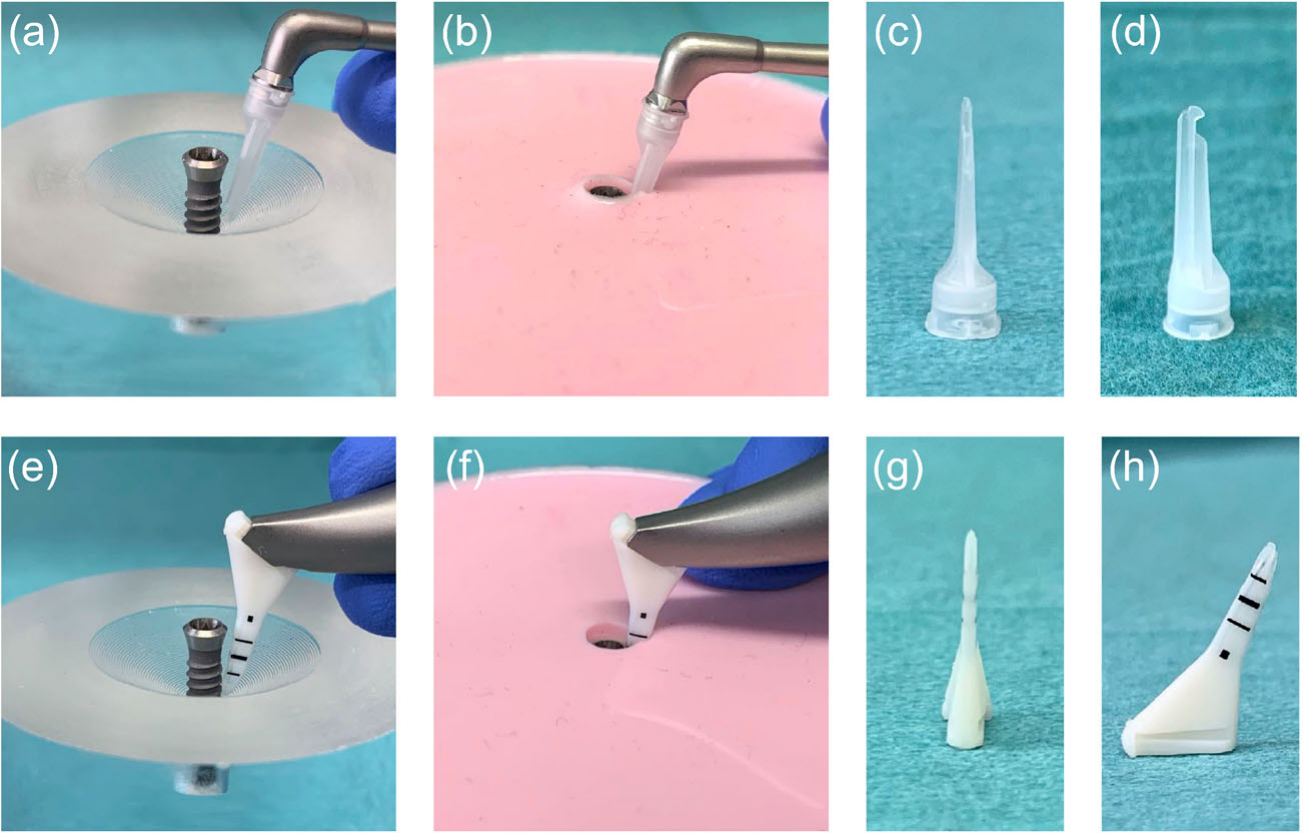
Use of both air-polishing devices (PERIO-FLOW^®^ by EMS, Perio-Mate by NSK) with the respective nozzle tip supplied by the manufacturer is shown: **a** Perio-Mate with attached Perio-Mate nozzle (NSK) in a surgical and **b** non-surgical approach, **c** frontal, **d** lateral view of Perio-Mate nozzle tip (NSK) with two outlets (1x towards the front and 1x towards the apical direction); **e** PERIO-FLOW^®^ with attached PERIO-FLOW^®^ nozzle tip (EMS) in a surgical and **f** non-surgical approach, **g** frontal, and **h** lateral view of PERIO-FLOW^®^ nozzle tip (EMS) with three outlets (1x towards the front and 2x opposite towards the side)

The in vitro defect model was computer-aided designed and manufactured in acrylic glass with 30° and 60° defect angulations as intraosseous defects and a 90° defect angulation as supraosseous defect (Figs. 1 and 2). The implants in the intraosseous defect simulation were placed 12 mm (complete rough implant surface) into the models in order to simulate a supracrestal position of the machined surface. The defect depth in the intraosseous defects was 6 mm. Therefore, the supraosseous defect simulation resulted in a supracrestal position of the machined and the rough implant surface [17–19]. Every implant was surrounded by acrylic glass in the apical 6 mm (Fig. 1). The defect models were uncovered to simulate the surgical/open approach and covered with an individually manufactured nontransparent mucosa mask (Adisil^®^ rosé 1:1, Siladent, Goslar, Germany) in order to prevent visual control of the cleaning procedure for the non-surgical/covered approach simulation (Fig. 3) [18].

### Simulation procedure

A total of 180 implants were examined. Each of the three different cleaning methods was tested on 20 of the 60 implants per defect angle. The 20 implants were again separated into 10 implants that were treated in a surgical approach and 10 implants that were treated in a non-surgical approach. Each of the three simulation models had a correspondingly individualized mucosal mask that was reused for all 30 implants that were cleaned in this model non-surgically. In the process, the following air powder abrasion and glycine powder type combinations (GAPA, glycine air powder abrasion) for surface decontamination were used (Fig. 3):

- GAPA1: Air-Flow® Handy 2+ (EMS GmbH, Munich, Germany) with a PERIO-FLOW^®^ handpiece (EMS) using glycine powder (3M™ Clinpro™ Glycine Prophy Powder, 3M Germany GmbH, Neuss, Germany) and an attached PERIO-FLOW^®^ nozzle tip (EMS) as frequently studied combination [29–32].
- GAPA2: Air-Flow® Handy 2+ (EMS) with a PERIO-FLOW^®^ handpiece (EMS) using glycine powder (AIRFLOW^®^ PERIO, EMS) and an attached PERIO-FLOW^®^ nozzle tip (EMS) as manufacturer combination
- GAPA3: Perio-Mate (NSK Europe GmbH, Eschborn, Germany) with glycine powder (Perio-Mate™ Powder, NSK) and attached Perio-Mate nozzle tip (NSK) using the medium ejection setting for powder and water spray volume as manufacturer combination.

Settings for the amount of water and powder emission were not freely selectable for GAPA1 and 2. Air pressure could not be adjusted on any of the selected devices. For each air-polishing device, combination settings were measured three times post-hoc with attached nozzle tip and then averaged: (1) water ejection [water was collected for 1 min and measured (ml/min)], (2) powder emission rate [the chamber of the air-polishing device was filled to the maximum, this amount was weighed and after 1 min of use it was weighed again and the difference was calculated (g/min)], and (3) the drive air-pressure [a multi-gauge was connected and read between the air-polishing device and the connection to the dental unit (bar)]. The nozzle tip for all combinations was used only once for every implant. All powder types had a mean particle size of ~ 25 μm and were water-soluble.

All implants were cleaned with or without mucosa mask for 2 min by the same operator (VT). Working distance and working angle were individually selected by the operator. After each instrumentation, the mucosa mask, if used, and the implant were removed. Dissolved color remnants were removed with a gentile air-water rinse for 10 s [17, 19].

### Photo documentation and analysis

**Fig. 4:**
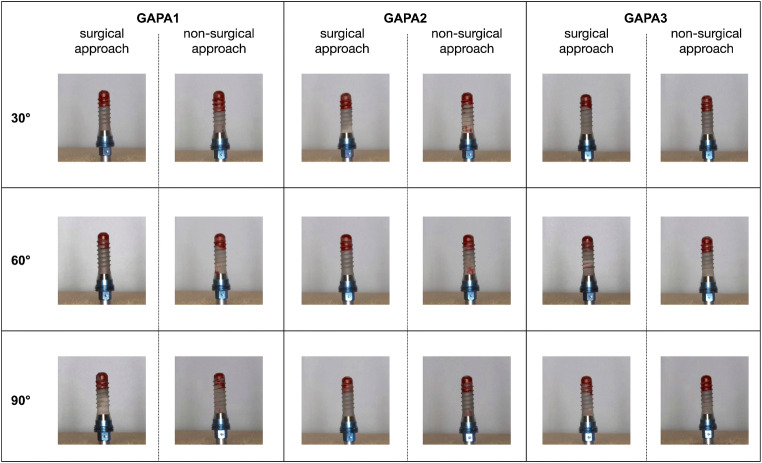
Detailed images of the cleaned surfaces according to treatment modality (GAPA 1–3) and defect angle

Photo documentation was performed in accordance with Sahrmann et al. [19] and Keim et al. [17]. Implants were removed individually without surface contact from the model and fixed (Implant Driver, OKTAGON Dental Implant System) in an individually fabricated immovable holder. Afterwards, both sides (180°) of the implants were digitally photographed in a standardized manner [(Canon EOS 70D, Tokyo, Japan) 31.4 cm distance, ISO 100, aperture f/32, exposure time 1/250 s) by the same examiner (VT) in an uniformly illuminated photo tent (proxistar, Kastl, Germany) with ring flash (Canon ring flash MR-14, Tokyo, Japan)] (Fig. 4) [17, 19].

All photos were analyzed (VT) using photo editing freeware (ImageJ 1.52a, U.S. National Institutes of Health, Bethesda, MD, USA; https://imagej.nih.gov/ij/). First a consistent region of interest (ROI) for each side of the implant was defined (HP). The ROI (output in pixels) corresponded to the implant surface of the respective site without the apical part (6 mm), which was included circumferentially in all models. Then, the relevant implant surface was, according to the photographed side, selected by overlay of the corresponding ROI. The remaining red color was marked within this area, and the number of pixels of the red color remnants was displayed. This number was multiplied by 100 and divided by the total number of pixels in the ROI resulting in a percentage (%). This procedure was repeated for the second side of the implant. The mean of both percentages was then calculated in order to obtain a total value of color remnants (%) of the whole selected implant surface. Basic values (brightness, contrast, sharpness) were not changed during the entire procedure.

**Fig. 5:**
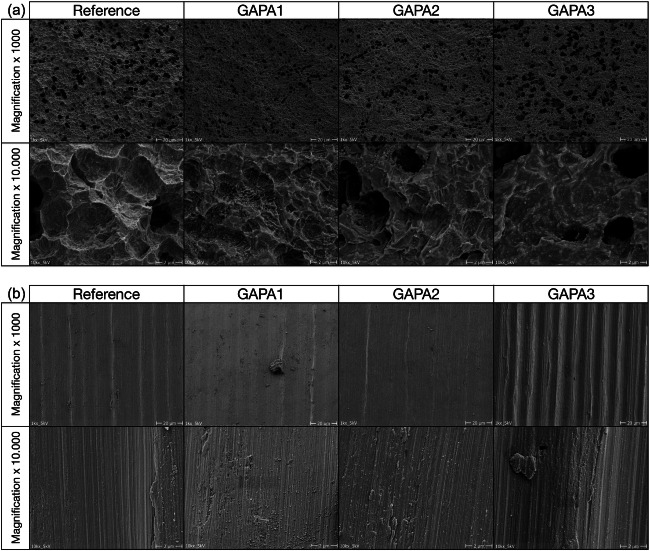
**a** Scanning electron microscopy images of untreated (reference) and non-surgically treated macro-threads of the implant surfaces by different instruments at magnification of × 1000 and × 10.000. **b** Scanning electron microscopy images of untreated (reference) and non-surgically treated machined collar of the implant by different instruments at magnification of × 1000 and × 10.000

**Fig. 6:**
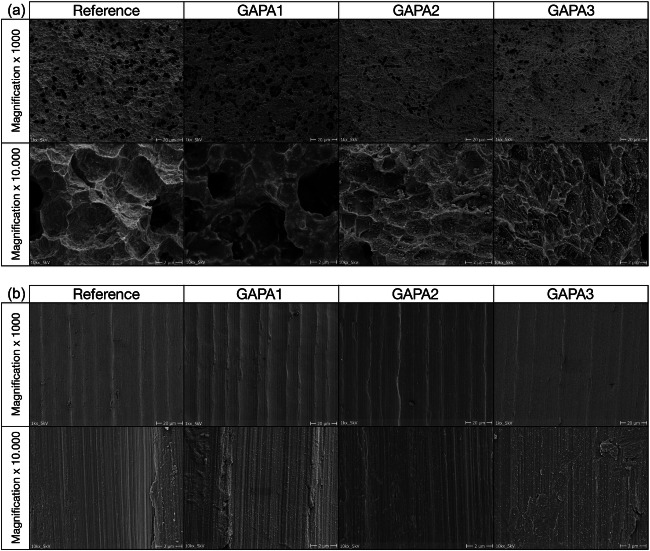
**a** Scanning electron microscopy images of untreated (reference) and non-surgically treated macro-threads of the implant surfaces by different instruments at magnification of × 1000 and × 10.000. **b** Scanning electron microscopy images of untreated (reference) and non-surgically treated machined collar of the implant by different instruments at magnification of × 1000 and × 10.000

In addition, SEM images (Philips XL 30 with lanthanum hexaboride cathode, 20kv, 10 mm distance, Philips, Amsterdam, The Netherlands) were exemplarily obtained after instrumentation of the machined and rough surfaces of one untreated (reference) and one non-surgically and surgically treated implant for each air-polishing method (GAPA1–3) (CR; magnification 1 × 1000 and 1 × 10,000) [17]. All samples were gold coated with a layer thickness of approximately 50 nm using an automatic sputtercoater (MSC2, KDF Electronic & Vacuum Services Inc., New Jersey, USA) (Figs. 5 and 6).

### Statistical evaluation

**Fig. 7:**
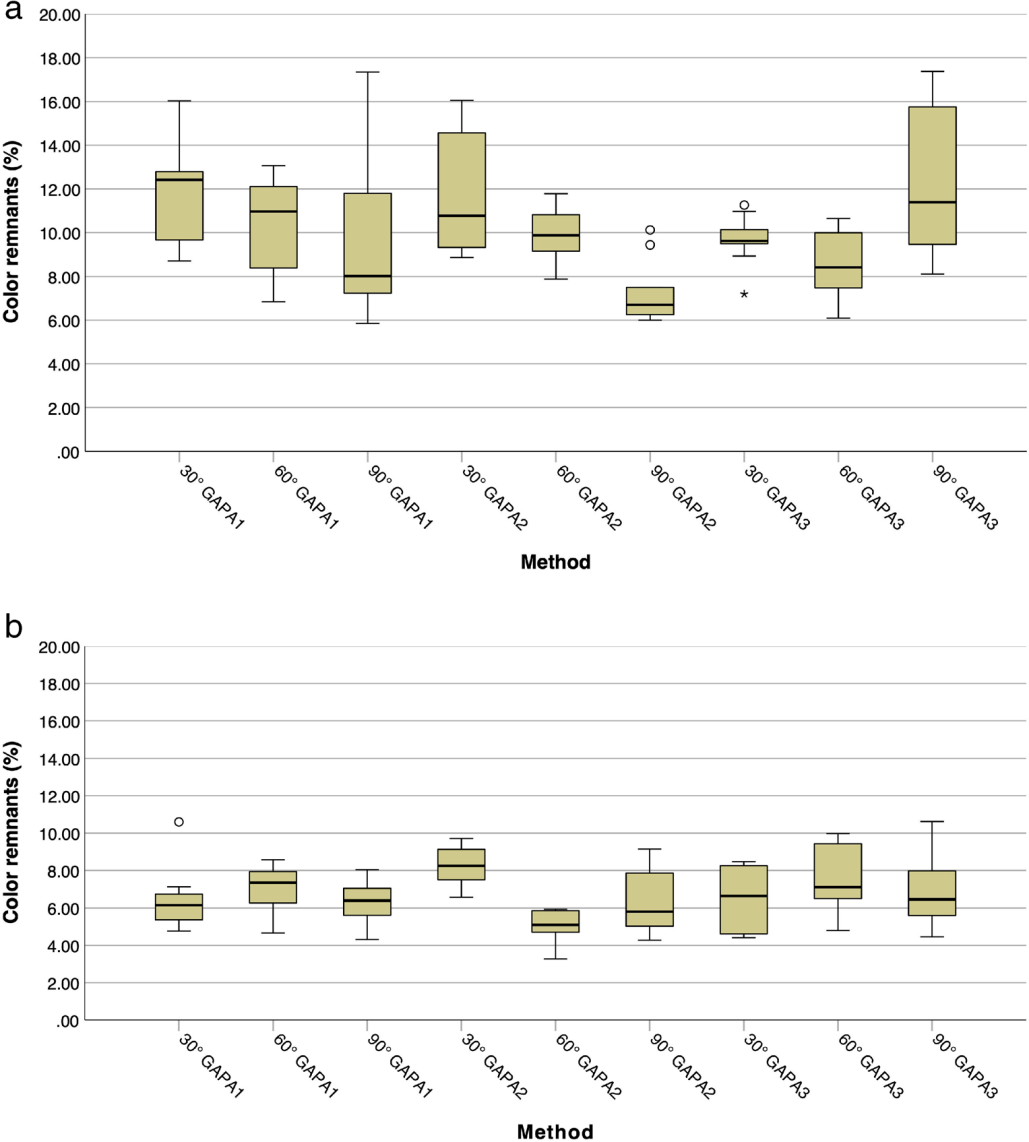
**a** Distribution of color remnants for different *non-surgical* treatment methods according to each defect angulation. **b** Distribution of color remnants for *surgical* different treatment methods according to each defect angle

**Fig. 8:**
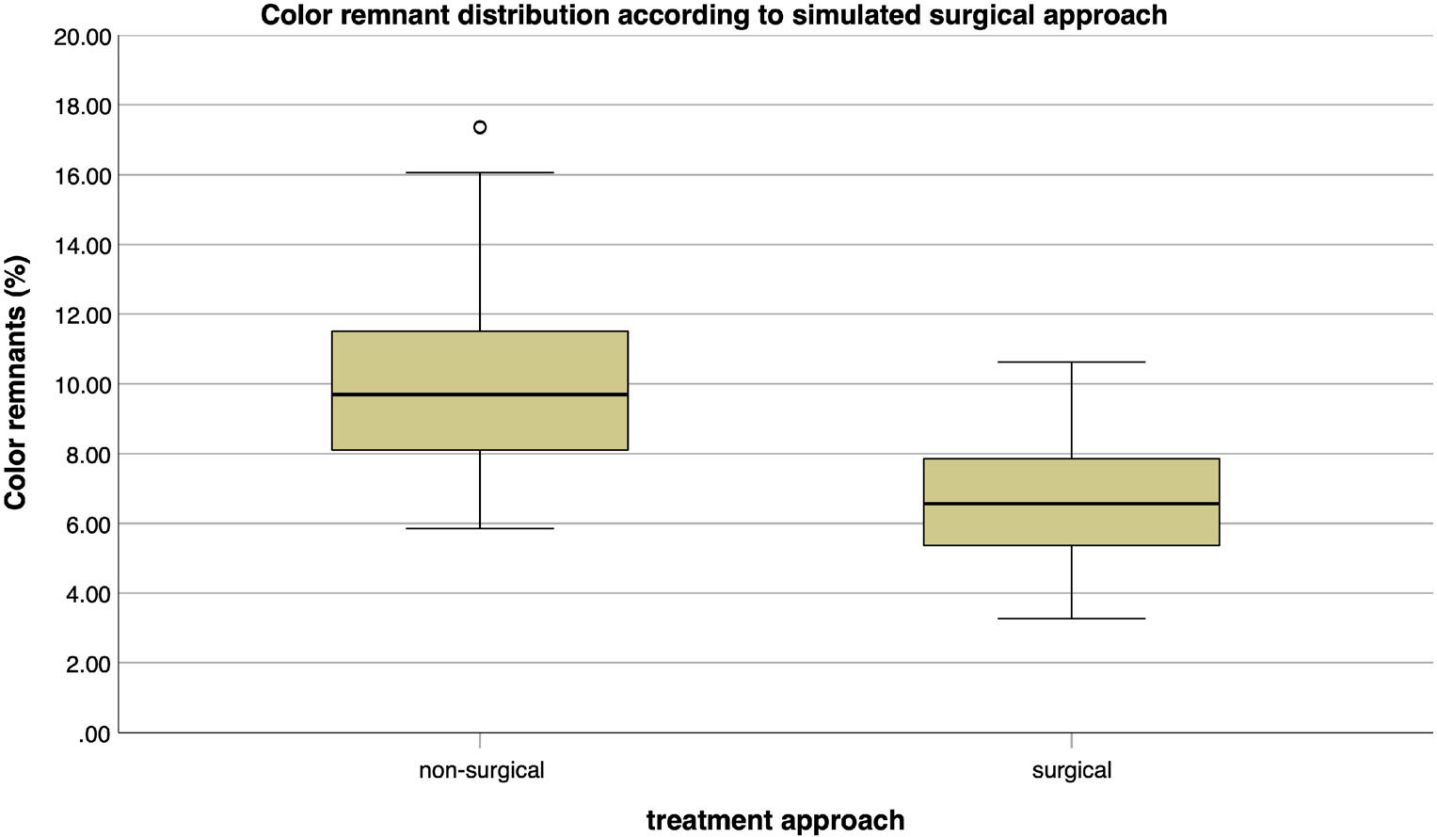
Distribution of color remnants according to each treatment approach

The statistical evaluation followed the protocol by Keim et al. [17]. The implant was considered as statistical unit. The percentage of non-cleaned surface was calculated for each implant (VT). First, the data were tested for normal distribution using the Kolmogorov-Smirnov test. Depending on this, descriptive data [mean value, medians, lower/upper quartiles, interquartile ranges (IR) and standard deviation] were analyzed for the cleaning methods and defect angulations according to the surgical or non-surgical simulation. Group comparisons were carried out using Kruskal-Wallis test for non-normally distributed data. The distribution of color remnants according to the different air-polishing device combinations (Fig. 7) and to the non-surgical and surgical approach (Fig. 8) are shown as box plot diagrams (wide black line, median; box, 25–75% range of all values; whiskers, range of all values without outliers; circle, outliers; asterisk, extreme outlier). By defining a *p* value < 0.001 as the significance level, multiple testing (Bonferroni correction [33]; 36 comparisons in the surgical or non-surgical group) was addressed. Group comparisons between the entirety of all surgical and non-surgical procedures were carried out by Mann-Whitney *U* test.

The statistical evaluation was carried out using the IBM^®^ SPSS^®^ Statistics 26 software package (IBM, Chicago, Illinois, USA).

The study was planned in compliance with the appropriate EQUATOR guidelines.

## Results

The results of the post-hoc testing of the device settings are shown in Table 1.

**Table 1:** Post-hoc testing of air-polishing device settings

Post-hoc testing of air-polishing device settings												
Air-polishing device combination	Water ejection (ml/min)				Powder emission rate (g/min)				Drive air-pressure (bar)			
	I	II	III	Mean ± SD	I	II	III	Mean ± SD	I	II	III	Mean ± SD
GAPA1	49.0	56.0	65.0	56.7 ± 8.02	3.54	3.51	2.26	4.28 ± 0.73	3.4	3.5	3.5	3.47 ± 0.06
GAPA2	60.0	65.0	63.0	62.7 ± 2.52	2.80	2.29	2.51	2.53 ± 0.26	3.5	3.6	3.5	3.53 ± 0.06
GAPA3	56.0	56.0	57.0	56.3 ± 0.58	3.86	4.82	3.81	4.16 ± 0.57	3.5	3.6	3.6	3.57 ± 0.06

SD = standard deviation

The overall comparison of the non-surgical approaches [mean ± standard deviation, 10.13 ± 2.75% (median, 9.68%; IR, 3.49)] with the surgical approaches [6.68 ± 1.66% (6.57%; IR, 2.53)] is significantly (*p* < 0.001) in favor of the surgical approach (Fig. 8). The box plot diagrams show no trend in cleaning efficacy regarding the applied air-polishing device combinations for either the non-surgical or the surgical approach (Fig. 7a/b). Nevertheless, the average smaller range of color remnants in Figs. 7b and 8 (range surgical approach: 3.27–10.62%) indicate a higher reproducibility of the cleaning results compared to the larger range (range non-surgical approach, 5.85–17.37%) in Figs. 7a and 8.

None of the investigated implant surfaces were completely free (0%) of color remnants. Residual color on the lower 6 mm of the implants was the result of a non-accessible area for cleaning because the implants were surrounded by the models in this part (Figs. 1 and 2).

The cleaning efficiency did not show any significant difference (*p* < 0.001) for each surgical or non-surgical cleaning method within each defect configuration (Table 2a/b). Within the individual air-polishing device combinations, there was only a significant difference between GAPA2 in the non-surgical approach (Table 2a) between 30° (median, 10.77%; IR, 5.48) and 90° (6.71%; IR, 1.77) and in the surgical approach (Table 2b) between 30° (8.25%; IR, 1.77) and 60° (5.10%; IR, 1.29).

**Table 2:** (a) Medians, means and standard deviations [%] as well as *p* values (Kruskal-Wallis) of residual colored surface areas after *non-surgical* treatment with three different air-polishing methods (GAPA1–3). Separate presentation for different treatment methods and defect angles. *N* = 10 for each treatment method in each defect angle. (b) Medians, means and standard deviations [%] as well as *p* values (Kruskal-Wallis) of residual colored surface areas after *surgical* treatment with three different air-polishing methods (GAPA1–3). Separately presentation for different treatment methods and defect angles. *N* = 10 for each treatment method in each defect angle

Defect angulation	30°					*p* value (30°–60°)	60°					*p* value (60°–90°)	90°					*p* value (30°–90°)
	Min. (%)	Max. (%)	Percentile (%)				Min. (%)	Max. (%)	Percentile (%)				Min. (%)	Max. (%)	Percentile (%)			
			25th	50th (median)	75th				25th	50th (median)	75th				25th	50th (median)	75th	
(a)																		
GAPA1	8.71	16.04	9.63	12.42	12.84	0.444	6.84	13.07	8.17	10.97	12.15	0.211	5.85	17.34	7.22	8.02	12.23	0.044
Mean ± SD (%)	11.78 ± 2.18						10.40 ± 2.11						9.51 ± 3.59					
*p* value GAPA1–2	0.823						0.788						0.148					
GAPA2	8.87	16.06	9.31	10.77	14.79	0.470	7.88	11.78	8.88	9.88	10.99	0.008	6.00	10.12	6.22	6.71	7.99	< 0.001
Mean ± SD (%)	11.77 ± 2.73						9.88 ± 1.32						7.25 ± 1.42					
*p* value GAPA2–3	0.321						0.192						0.003					
GAPA3	7.20	11.26	9.35	9.62	10.34	0.234	6.09	10.64	7.38	8.42	10.08	0.048	8.11	17.37	9.21	11.39	16.16	0.431
Mean ± SD (%)	9.69 ± 1.12						8.52 ± 1.49						12.39 ± 3.66					
*p* value GAPA1–3	0.224						0.115						0.118					
Overall*	7.20	16.06	9.51	10.25	12.71	0.048	6.09	13.07	8.00	9.88	10.91	0.398	5.85	17.37	7.05	8.35	11.94	0.005
	11.08 ± 2.28						9.60 ± 1.80						9.72 ± 3.65					
(b)																		
GAPA1	4.76	10.60	5.25	6.16	6.83	0.430	4.66	8.57	6.25	7.35	7.98	0.363	4.31	8.04	5.34	6.39	7.20	0.904
Mean ± SD (%)	6.40 ± 1.68						7.10 ± 1.16						6.24 ± 1.23					
*p* value GAPA1–2	0.144						0.038						0.968					
GAPA2	6.56	9.71	7.44	8.25	9.21	< 0.001	3.27	5.94	4.57	5.10	5.86	0.202	4.28	9.15	5.02	5.81	8.05	0.049
Mean ± SD (%)	8.26 ± 1.02						5.02 ± 0.84						6.33 ± 1.70					
*p* value GAPA2–3	0.134						0.019						0.591					
GAPA3	4.4.1	8.47	4.58	6.63	8.26	0.362	4.80	9.97	6.29	7.10	9.45	0.552	4.46	10.62	5.39	6.46	8.18	0.751
Mean ± SD (%)	6.49 ± 1.74						7.45 ± 1.71						6.86 ± 1.93					
*p* value GAPA1–3	0.969						0.788						0.565					
Overall*	4.41	10.60	5.50	7.18	8.33	0.370	3.27	9.97	5.17	6.38	7.72	0.370	4.28	10.62	0.05	6.34	7.82	0.370
	7.05 ± 1.70						6.52 ± 1.66						6.48 ± 1.61					

*The comparison between the overall non-surgical and surgical approach was significantly different for each angulation (*p* < 0.001)

Consequently, for the majority of all comparisons within the various air-polishing device combinations and within the three different defect angulations, no differences in cleaning efficacy were shown. The null hypothesis was rejected.

All SEM images confirm the complex surface morphology of the selected implant system. Cleaning with air-polishing devices showed no serious surface damage in the obtained SEM images, neither in the area of the machined nor in the area of the rough implant surface (Figs. 5 and 6). The machined rings were smoothened after air-polishing indicating a slight modification of the surface.

## Discussion

The aim of this study was to compare the efficacy of three different combinations of air-polishing methods for implant surface decontamination in different non-surgical and surgical in vitro defect models (30°, 60°, 90°). Overall, the surgical approaches were significantly superior (*p < 0.001*) to the non-surgical ones. The low color remnant value for all methods and approaches indicates a good cleaning efficacy, even if no implant could be completely freed (0%) from the color. Among the three investigated methods (GAPA1–3), no significant differences occurred. Only GAPA2 showed significant differences (*p < 0.001*) within the methods in the non-surgical (30°–90°) and surgical procedure (30°–60°). The null hypothesis was rejected.

The main reason for the use of nozzle tips in the non-surgical as well as in the surgical approach was the comparability to other studies [17, 19]. In addition, peri-implant defects are typically shaped around implants in spite of flap mobilization and, depending on the depth, a nozzle tip may still facilitate better accessibility.

The authors want to anticipate that, according to the current state of affairs, the surgical application of air-polishing devices represents an off-label use due to the lack of sterility, which is why an in vitro investigation was initially carried out.

As Petersilka in 2011 described [34], air-polishing with its powder-water ejection is subject to the so-called ricochet effect, which may have an influence on the cleaning efficacy. For particles that hit a hard surface, this effect describes an uncontrolled rebound, bounce, or skip off a surface depending on its texture. The fact that there were no significant differences within the respective defect angulations suggests that neither the powder used nor the geometry of the nozzle applied have a major influence on the results achieved. Also, the defect geometry obviously no longer plays a major role in the uniform use of air-polishing devices. Only the GAPA2 combination showed significant differences (*p < 0.001*) in cleaning efficiency (non-surgical, 30°–90°; surgical, 30°–60°). On the one hand, this may be due to the narrow defect angle of 30° involved in both cases, which may be more difficult to access. On the other hand, the slightly different post-hoc tests of the device settings of GAPA2 compared to GAPA1 and GAPA3 could have influenced this.

The superiority of air-polishing methods [35], but especially of glycine-based air-polishing devices, over the use of rigid instruments (e.g. steel curettes, sonic scaler) with regard to cleaning efficacy and less surface damage in non-surgical/covered and surgical/open in vitro models has recently been repeatedly proven [17, 18, 25, 36]. Therefore, the present in vitro study deals with the efficacy of different glycine-based air-polishing methods in two simulated treatment approaches (non-surgical/covered and surgical/open).

With regard to the non-surgical procedure, 20 implants per angulation were cleaned with glycine-based air-polishing in a very similar study [18]. The implant systems used were also very similar in micro and macro structure. However, the results are significantly different, when considering both the data based on the use of the same instruments (GAPA2) and the overall average data. While Ronay et al. [18] found 40.15 ± 10.40% (30°), 40.30 ± 7.12% (60°), and 21.20 ± 8.96% (90°) color remnants, the average values of our investigation were 11.08 ± 2.28% (30°), 9.60 ± 1.80 (60°), and 9.72 ± 3.65% (90°).

Regarding the surgical procedure, the comparison to Sahrmann et al. [19] and Keim et al. [17] can be made, who also used similar experimental setups. The former investigation reports 16.1 ± 3.7% (30°), 12.7 ± 2.8% (60°), or 5.0 ± 1.4% (90°) color remnant, and Keim et al. [17] showed color remnants in 8.03 ± 2.43% (30°), 0.13 ± 0.26% (60°), and 0.58 ± 0.88% (90°) on the corresponding implants. The present study found on average 7.05 ± 1.70% (30°), 6.52 ± 1.66% (60°), and 6.48 ± 1.61% (90°) color remnants on the implant surfaces after the surgical/open approach.

In the non-surgical comparison, a different implant system was used in this study than in Ronay et al. [18]. Both implants are similar with a machined implant shoulder (Ronay et al. [18], 1.0 mm; present study, 1.8 mm) and a rough part underneath with macro-threads at quite similar distances (Ronay et al. [18], 1.0 mm; present study, 1.25 mm) and with the same thread depth (0.35 mm). Nevertheless, the two systems differed in their surface properties. The surface roughness of the sand-blasted thermal acid-etched implants used by Ronay et al. [18] is *R*_a_ = 2.35 μm. In the present study, the sand-blasted and acid-etched implants had an average roughness of *R*_a_ = 1.69 μm. This different surface roughness may partially explain the different results. Due to the rougher surface in Ronay et al. [18], the adhesion of the color used may have been more pronounced than in the present study and therefore was more difficult to remove. Another important difference between the two studies is that Ronay et al. [18] carried out the procedure randomly, while there was no randomization in this study and only one examiner carried out all cleaning procedures and analyses.

Both studies used mucosa masks made from different materials, so that the rigidity and thus the resistance to the instruments inserted between the implant and the mask were different. The width of the bone defect models and thus the thickness of the mucosal mask may play an important role, which was not specified in either study. The thicker it was, the less firmly it was pressed onto the implant surface during cleaning (while it was held by the examiner). Too much pressure could position the respective nozzle incorrectly and the cleaning effect could suffer. The adaptation of the mucosal mask on the model was identical (it was just put over the model). Furthermore, the material used for the mucosal mask is different. Ronay et al. have used a material more similar to human tissue (ballistic gelatine), but in combination with the above-mentioned pressure the effect cannot be clearly distinguished.

Looking at the surgical/open procedure, it can be seen that the results in these studies overall are closer together [17, 19]. Nevertheless, differences can also be found here. The implants were quite similar in macroscopic structure (Sahrmann et al., [19]: thread distance 1.25 mm, thread depth 0.35 mm, machined collar 1.8 mm; Keim et al. [17]: thread distance 0.6 mm, thread depth 0.32 mm, machined collar 1.5 mm; present study: thread distance 1.25 mm, thread depth 0.35 mm, machined collar 1.8 mm). However, the study with the highest color remnant values used the implant system with the highest surface roughness (Sahrmann et al. [19]: 2.93 μm; Keim et al. [17]: 0.76 μm; present study: 1.69 μm; values were presented by Sammonos et al. [37]). The results of Keim et al. [17] and this study are less different, which may be due to the fact that each implant was used only once in both studies. Sahrmann et al. [19] have used the implants several times, which could possibly have influenced their surface roughness/texture [38].

In general, the studies by Ronay et al. [18] and Sahrmann et al. [19] differ from those of Keim [17] and the present investigation in that no uniform method for analyzing the color remnants was applied. In addition, the device settings (drive air-pressure, water ejection, powder emission) as well as the depth, the design, and movement of the nozzle play an important role in the defect to be cleaned [39]. The device settings were measured post-hoc, but cannot be compared because they are not reported in the other studies [17–19]. The nozzle used in the combinations GAPA1 and GAPA2 has more stable walls than the nozzle from the combination GAPA3 and three outlet openings (2x laterally, 1x vertically) for the powder-water jet mixture, while the nozzle from the GAPA3 combination has two outlet openings (1x laterally, 1x vertically). With the GAPA1 and GAPA2 nozzle, the powder-water jet mixture hits the implant surface directly through a side outlet, while with the GAPA3 nozzle it does not directly hit the implant surface but emerges laterally from it. With the GAPA3 nozzle, a mixture is not ejected; instead, water and powder are guided separately within the nozzle. The water finally emerges vertically, and the powder emerges laterally. Due to its slim design, the GAPA3 nozzle rotates laterally when performing the non-surgical procedure, as soon as it is inserted into the pocket. This is not the case with the nozzle used in the GAPA 1 and GAPA2 combination due to its higher rigidity.

In general, various systematic reviews [9, 11, 21] as well as recent clinical studies [23, 40] show advantageous results in the use of glycine-based air-polishing devices for the (non-)surgical treatment of peri-implantitis. However, their ability to restore the biocompatibility of the implant surface [41] and to maintain achieved results in the long term [42] are questioned. Assuming that more effective surface decontamination would also mean clinically better results, then air-polishing would probably lead to improved therapy results. However, the extrapolation of these in vitro results to the clinic is not allowed.

The present study presupposed that removal of the suprastructure from the implants for a more straightforward accessibility of the implant surface is possible. Otherwise poorer results due to the limited accessibility may have been achieved [43]. In everyday clinical practice, missing removability of a cemented suprastructure should be considered.

SEM images of this study confirm other investigations showing only slight changes of implant surface morphology after application of glycine-based air-polishing devices [17–19, 26–28, 41].

A current review on the in vitro efficacy of air-polishing devices on titanium implant surface damages concludes that they are less damaging compared to harder and larger-sized powders such as sodium bicarbonate especially when using glycine-based powder types [41]. These results are confirmed by recent in vitro [26, 27] and in vivo [28] studies. On the other hand, there are indications that coarser powder types can achieve a higher cleaning efficacy than finer ones [27]. Although the ability of glycine-based powders to maintain biocompatibility when applied to titanium surfaces is questioned [41], it still appears more likely than with major damages such as, e.g. caused by steel curettes [17–19].

A limitation of the present study is biofilm imitation by use of color without simulation of further oral cavity-specific influences. Nevertheless, this type of in vitro model and the use of color as “artificial plaque” have prevailed [17–19, 25]. Further, the rigidity and re-use of the mucosa mask differ from the oral mucosa which may lead to results different from the oral cavity. Photographic analysis from a single angle may cause less accurate differentiation of color remnants on the apically and coronally facing site of the threads than in previous investigations [16, 25]. The fact that one examiner (VT) did both the implant cleaning and the subsequent analysis without randomization may lead to bias.

In summary, the results of this in vitro study show that the use of glycine-based air-polishing devices, regardless of the manufacturer and largely also of the defect geometry, achieves in a non-surgical/covered and surgical/open approach a high cleaning efficacy according to the chosen implant system. Nevertheless, a complete surface decontamination was not achieved with any device.
